# Adherence to Quality Indicators for Breast Cancer Management in a Multidisciplinary Training Program

**DOI:** 10.3390/jpm13121693

**Published:** 2023-12-08

**Authors:** Maria Grazia Baù, Fulvio Borella, Maria Piera Mano, Livia Giordano, Marco Carosso, Alessandra Surace, Aurelia Mondino, Niccolò Gallio, Chiara Benedetto

**Affiliations:** 1 Gynecology and Obstetrics Unit, Maria Vittoria Hospital, 10144 Turin, Italy; mariagrazia.bau@gmail.com; 2 Gynecology and Obstetrics Unit 1, AOU Città della Salute e della Scienza di Torino, Sant’Anna Hospital, 10126 Turin, Italy; marco.carosso@unito.it (M.C.); chiara.benedetto@unito.it (C.B.); 3 Department of Surgical Sciences, University of Turin, Via Ventimiglia 1, 10126 Turin, Italyaureliamondino@gmail.com (A.M.); 4 Unit of Epidemiology, CPO Piemonte, AOU Città della Salute e della Scienza di Torino, San Giovanni Antica Sede, 10123 Turin, Italy; 5 Gynecology and Obstetrics Unit, Michele e Pietro Ferrero Hospital, 12060 Verduno, Italy; alessandra.sur@gmail.com; 6 Gynecology and Obstetrics Unit 2, AOU Città della Salute e della Scienza di Torino, Sant’Anna Hospital, 10126 Turin, Italy; niccolo.gallio@edu.unito.it

**Keywords:** aesthetic, breast cancer, breast unit, quality indicators, functional results, management, waiting time

## Abstract

**Background**: The management of early breast cancer (BC) needs supervision and skill maintenance, and should be performed by specialists working as a team in multidisciplinary breast units. This approach aims to improve the long-term survival and quality of life of patients with BC. **Methods**: This was a prospective observational study including patients newly diagnosed with operable BC. The study encompassed the pre-surgical phase, throughout the diagnostic and surgical workout, and included post-therapeutic master multidisciplinary team meetings (MTMs) sessions, between 2019 and 2022. **Results**: We enrolled 280 patients with BC from eight breast units. The Senonetwork indicators regarding diagnosis, waiting time, loco-regional treatment, and adjuvant therapy were collected for each patient discussed. **Conclusions**: Overall, the majority of quality indicators were respected among breast units. The most critical issue referred to timing indicators: more than 30 days from MTM to surgery, more than 42 days from diagnosis to surgery, and more than 60 days from the first screening mammogram to surgery for many patients. Some aspects of the histopathological diagnosis of intraductal BC also need to be improved. Furthermore, other critical issues in our study regarded some aesthetical indicators, demonstrating low interest in these essential quality indicators.

## 1. Introduction

Early breast cancer (BC) management requires continuous supervision and skill maintenance by specialists working in multidisciplinary breast units, to optimize the diagnosis, treatment, and follow-up of patients and improve the long-term survival and quality of life of BC patients [1]. Specialists from different fields, such as surgery, medical oncology, radiation oncology, radiology, pathology, nuclear medicine, and nursing, need to work together in a coordinated and collaborative manner to provide the best care for each patient [2]. Indeed, according to the existing data, BC patients can benefit from better treatment in breast units with a multidisciplinary team compared with those treated in non-specialized facilities, as they improve the survival rate and quality of life [2]. Therefore, BC referral centers should undergo regular internal and external quality-control procedures to provide patients with the highest standard of care [3]. In Europe, the European Society of Breast Cancer Specialists (EUSOMA) sets the requirements for referral BC centers and promotes the voluntary European certification process to facilitate compliance with the recognized guidelines. EUSOMA published a position paper in 2010 with 17 quality indicators for BC centers, which was updated in 2017 to incorporate new knowledge regarding BC treatment [4,5]. Similarly, the Italian Society for BC quality control (Senonetwork) published a set of benchmark quality indicators in 2013 to standardize auditing and quality assurance, and to establish an agreed minimum standard of care [6]. In this context, multidisciplinary team meetings (MTMs) are a widely accepted and well-established instrument to discuss the best management for BC patients [7,8,9]. MTMs have been shown to improve the quality and outcomes of BC care, such as reducing the number of unnecessary surgeries, increasing the use of breast-conserving surgery, enhancing adherence to treatment protocols, and improving the survival and quality of life of patients [10,11,12]. MTMs also provide a platform for education, research, and audit, as well as an opportunity for patient involvement and advocacy [10,11,12]. However, MTMs require a significant investment of time, resources, and expertise, and depend on the availability and participation of core members, quality and completeness of data, structure and organization of the meeting, leadership and communication skills of the chairperson, and evaluation and feedback mechanisms. In Italy, BC care is provided by different institutions, namely the Scientific Institute for Research, Hospitalization, and Healthcare; academic or local hospitals; and private clinics. To guarantee adherence to national guidelines and standardization of cancer management, the Italian healthcare minister introduced the Oncological Network [13], a system that coordinates and supervises cancer treatment across the country. The Oncological Network also promotes the use of quality indicators to monitor and evaluate the quality of care for BC patients [9]. For BC, the quality indicators proposed by Senonetwork [14] are registered and monitored through a digital system “QT-breast”. In BC care centers, MTMs are a good instrument to ensure quality control processes.

In Piedmont, a northwest Italian region, there are 16 breast units that are active and inter-connected through the Oncological Network, collaborating closely with the regional BC screening program. In this region, breast unit specialists must attend a post-graduate specialization Master’s course within a permanent interactive program called FIM (interactive multidisciplinary formation). This program trains BC specialists on how to manage BC patients according to guidelines in a constant and permanent way. According to recent publications, adherence to EUSOMA 22 quality indicators improves the quality of care for BC when achieving the minimum standard of care (12/13 quality indicators) [15,16]. Given these premises, we conducted a prospective multicenter analysis of quality of care in the early BC pathway in real-life access to cancer care including all institutions whose specialists attended the BC formation program in the largest Oncological Network region. We evaluated medical doctors’ adherence to Senonetwork quality indicators [14] in 280 patients affected by BC in a shared masterclass MTM setting, using medical information from QT-breast medical records. We then conducted an analysis of quality indicators in BC care at a regional level, as reported by each specialist. We evaluated whether these quality indicators aligned with a set of high-quality indicators established by Senonetwork.

## 2. Material and Methods

We conducted a prospective observational study between 1 January 2019 and 1 January 2021 including patients newly diagnosed with early BC, and discussed the pre-surgical phase, the period during diagnostic and surgical workout, and the post-therapeutic master MTMs. The study was part of a regional program for formation and skill maintenance directed toward breast-unit operating doctors from all breast specialties (radiologists, pathologists, surgeons, gynecologists, oncologists, radiotherapists, and primary care). The 2-year master consisted of practical and theoretical lessons involving 10 attendees. During the master, each attendee had to present and discuss 35 consecutive-treated cases and report each case’s quality indicators according to Senonetwork indications. Patients were treated by 8 public state hospitals. Then, we built the database prospectively and collected data directly from institutions during the master timetable using digital medical records.

The collected data were presented as absolute counts, percentages, and ranges.

## 3. Results

We reported cases in the dataset from eight breast units with an average of 300 patients treated/year (range 78–969), of which the average number of screening-detected patients was 120 (range 25–377) in Piedmont; all data were anonymized. Each breast unit reported data from 25 invasive BCs and 10 intraductal BCs (N = 280).

### 3.1. Diagnosis

All breast units performed a definitive pre-operative diagnosis of invasive cancer for these cases. The following information was reported for all invasive BCs: histological type, grading, hormonal receptors, HER2, Ki67 expression, presence of peritumoral vascular invasion, stage, and pathological dimension. Regarding intraductal BC, all breast units reported the histological type and presence or absence of comedogenic necrosis.

The following indicators were not met by all breast units: tumor grading (5/8 breasts met this indicator, total 91%, minimum standard ≥ 90%), pathological tumor dimension reported in the definitive histological examination (5/8 breasts met this indicator, total 83%, minimum standard ≥ 90%), and minimum distance from the free margin reported in the definitive histological examination (4/8 breasts met this indicator, total 61%, minimum standard ≥ 90%). Preoperative magnetic resonance imaging was performed for 92/200 cases (46% of patients with invasive cancer, the minimum standard of 5% was achieved by all centers involved (range 12–100%)). X-rays of the surgical specimen in two perpendicular projections were performed in 134/173 (77%) patients (5/8 breasts met this indicator, minimum standard ≥ 90%, range 0–100%). Data about diagnosis quality indicators are available in Table 1.

### 3.2. Waiting Time and Treatment

The waiting time of 30 days from the surgical indication to surgery was met by 209/280 cases (74%, four breast units). The indicator of 42 days since the first diagnostic examination was met by 173/280 cases (62%, three breast units). Finally, the indicator of a 60-day interval from the screening mammography to surgery was met by 125/280 cases (44%, one breast unit). Overall, the indicators regarding breast and axillary surgery and adherence to adjuvant treatments (radiotherapy, hormone therapy, chemotherapy, and anti-HER2 therapy) were respected. For details, see Table 2.

### 3.3. Aesthetic and Functional Quality Indicators

Here, 228 cases (81%) showed no retracting or diastasis of the surgical scar; this quality indicator was met in six hospitals. No skin discoloration was observed in 258 patients (92%). Among the patients who underwent breast conservative surgery, approximately 50% had a degree of asymmetry of the nipple–areola complex compared with the contralateral one.

Nipple-sparing or skin-sparing mastectomy was performed in 23 patients (59%) and immediate reconstruction in 13 patients (56%); however, for some quality indicators, the minimum standard was not globally achieved: immediate reconstruction without direct contact with the prothesis and flap (84%, 3 breast units met this quality indicator), the use of the acellular dermal matrix (39%, 2 breast units met this quality indicator), and lost implantation at 6 months from the immediate reconstruction (13%, 5 breast units met this quality indicator). Homolateral axillary lymphedema after axillary dissection was observed in two cases (15%). Homolateral axillary lymphedema after lymph node biopsy was present in only one case (%). Articular limitation on the homolateral shoulder of 10% was reported in 13 cases (5%). Data on aesthetic and functional quality indicators are available in Table 3.

## 4. Discussion

BC is the most common cancer among women worldwide, and it poses a significant challenge for healthcare systems. The management of BC requires a multidisciplinary approach that involves different specialists and interventions. To ensure the quality and effectiveness of breast cancer care, it is essential to monitor and evaluate the processes and outcomes of the services provided. There are three distinct types of quality indicators: structure indicators, which assess the sources utilized in service provision; process indicators, which evaluate the actions performed during patient care; and outcome indicators, which analyze the results of patient care.

Patient-centered care and shared decision making—which involves clinicians and patients collaborating to share evidence, consider options, and make care decisions based on choices and beliefs—have become increasingly significant in recent years. However, quality indicators and their reference values are decided by a consensus and could change over time, making it difficult to distinguish between mandatory and ancillary indicators [17].

Regarding the perioperative diagnostic procedure, the data collected by eight physicians participating in the program show that histological evaluation is usually reported according to the criteria required by Senonetwork, especially for invasive BC; however, the reporting of the distance of margins from intraductal areas emerged as a critical issue that needs improvement. Overall, even reporting of the size of intraductal tumors is an indicator that has not always been adhered to.

This is partly due to the challenges of identifying some histological features and the status of the pathological margins of intraductal carcinoma, which can vary depending on the sampling and processing methods. Therefore, several systems have been proposed to improve the classification and grading of intraductal carcinoma, but their reproducibility and reliability among pathologists are not always satisfactory [18]. To overcome these limitations, new tools are being developed and tested to enhance the performance of histopathological analysis, such as machine-learning technologies that can automate and standardize the assessment of intraductal carcinoma. These technologies can be especially useful in settings where histopathological diagnostics can be problematic due to a lack of resources or expertise [19].

Over 50% of patients did not undergo preoperative magnetic resonance imaging, but, overall, the indicator was met by all centers (>5%). However, not all BC patients need magnetic resonance imaging during the diagnostic process; the indication should be limited to specific cases (e.g., some intraductal carcinoma, lobular invasive cancer, advanced stage, Paget disease, or chest wall tumors) [20]. One of the challenges of breast-conserving surgery is to ensure that adequate margins are obtained while preserving the cosmetic appearance of the breast. This requires an accurate assessment of the surgical specimen, which can be achieved through radiological or pathological methods. Radiological methods involve taking X-rays of the specimen to check for residual tumors. However, this may not be feasible in some settings, especially if there is no dedicated radiologist available or if there is a lack of equipment; in our study, three breast units did not meet this quality indicator. To overcome this limitation, several devices have been developed to evaluate the margins intraoperatively—which means during surgery [21]. One of the most widely used devices is Faxitron© (Hologic^®^, Marlborough, MA, USA, which is a portable X-ray machine that can be placed in the operating room. Faxitron© allows the surgeon to take X-rays of the specimen and view them on a monitor within minutes. This can help reduce the need for re-excision, which is a second surgery to remove more tissue if positive margins are found. Faxitron© has been shown to be cost effective, easy to use, and reliable for margin assessment [22,23,24].

The most critical issue we observed concerned waiting time quality indicators: waiting more than 30 days from MTM to surgery, more than 42 days from diagnosis to surgery, or more than 60 days from the first screening mammography. Exceeding these waiting times could potentially expose patients to prognostic risks, meaning it could negatively affect the outcome of their disease. The reasons for these delays can vary. One recent factor that may have contributed to longer waiting times is the SARS-CoV-2 pandemic.

A decrease in surgical activities was, in fact, observed in one of the eight institutions analyzed (Breast Unit of Città della Salute e della Scienza, Turin) during the SARS-CoV-2 pandemic [25,26]. This decrease can be attributed to a delay in screening and patients’ fear of accessing hospitals for routine diagnostic procedures and symptom checkup [25,26].

However, it is important to note that the impact of the pandemic on waiting times has been inconsistent across different studies and locations. Some well-organized breast units, globally, did not report a significant increase in waiting times during the pandemic [27,28,29,30]. This suggests that factors other than the pandemic can also play a role in these delays.

These findings should not be overlooked, as an excessive delay between diagnosis and treatment can have a negative impact on prognosis: a recent study found that surgery 6 weeks after diagnosis compared with surgery 3 weeks after diagnosis conferred a 1.26-fold increased hazard rate of death from all causes [31].

The reintervention rate (quality indicator failed in 2 out of 8 for intraductal carcinoma) is consistent with data reported in the literature [32], being higher among in situ lesions (perhaps because of difficulties in surgical margin interpretation). All quality indicators about breast and axillary surgery were respected.

Overall, these data demonstrate the good quality of BC management among the centers involved in this study. Regarding adjuvant treatment, only five patients did not receive hormone therapy, because they refused it. All quality indicators about adjuvant treatment were respected.

Another critical issue in our study was related to some aesthetic quality indicators, showing a low interest in these essential quality indicators.

This problem could stem from various issues in our country. A recent multicenter prospective study conducted on 6515 women from 10 Italian Senonetwork breast centers showed a lack of measurable, reproducible, and validated aesthetic outcome indicators to monitor their performance; furthermore, similar to the centers that participated in this study, the aesthetic and functional outcomes were likely not rigorously considered [33]. The authors suggested some simple parameters to improve the aesthetic and functional indicators: performing photo acquisition and storage for all patients, monitoring the proportion of skin-sparing/nipple-sparing mastectomies out of the total mastectomies, and evaluating the symmetry of the nipple–areola complex. Our data also suggest the need for complementary recommendations.

This study has some strengths and limitations. The prospective design enabled the acquisition of better-quality data. In addition, further support for the quality of the collected data concerns the multidisciplinary discussion within the master of all the cases collected. A limitation is that the results might not reflect the national scenario, as they only represent the experience of eight Italian breast units reported by master students.

## 5. Conclusions

From our study, it emerges that the Senonetwork aesthetics quality indicators are not always met and guaranteed. The recent introduction of oncoplastic techniques, for both conservative and less conservative BC surgery, requires an effort to maintain regular control of the quality of treatment. Adherence to recognized and validated indicators and periodic analysis of this adherence could help to improve the overall quality of the treatment offered. The choice of the oncoplastic type of surgery should be discussed for each patient in a multidisciplinary team and preferably be based on both objective and subjective criteria. The main objectives of oncoplastic surgery are to improve patient satisfaction and, consequently, quality of life, to ensure treatment efficacy along with an optimal aesthetic result, as well as to minimize the negative psychological impact of radical surgery such as mastectomy and nipple removal. However, unlike other domains of BC care, there are no agreed, measurable, reproducible, and validated indicators of aesthetic outcomes after breast surgery, which limits the monitoring and benchmarking of each breast center’s performance. Regarding the other quality indicators that have not been respected by all of the breast units, technology development could help in improving a pathological intraductal evaluation, therefore meeting these quality indicators. The waiting list issue could also be improved by increasing healthcare system investment, especially after a pandemic. Our study has identified some critical issues regarding the application of quality indicators that must be considered to improve the care of patients with BC. It could also be helpful to compare our data with other settings and countries, and to understand if there are any differences in the application of the quality criteria.

## Figures and Tables

**Table 1:** Senonetwork indicators regarding diagnosis among 8 breast units. The indicators below the minimum quality threshold are in bold.

		Breast Unit									
Diagnosis	Total	1	2	3	4	5	6	7	8	Minimum Standard	Range
Breast cancers (invasive or intraductal) with a definitive pre-operative diagnosis (C5 or B5) (N) (%)	280/280 (100%)	35 (100%)	35 (100%)	35 (100%)	35 (100%)	35 (100%)	35 (100%)	35 (100%)	25 (100%)	≥80%	100%
Invasive cancer cases with histological type N (%)	200/200 (100%)	25 (100%)	25 (100%)	25 (100%)	25 (100%)	25 (100%)	25 (100%)	25 (100%)	25 (100%)	≥90%	100%
Invasive cancer cases with grading N (%)	200/200 (100%)	25 (100%)	25 (100%)	25 (100%)	25 (100%)	25 (100%)	25 (100%)	25 (100%)	25 (100%)	≥90%	100%
Invasive cancer cases with hormone receptors N (%)	200/200 (100%)	25 (100%)	25 (100%)	25 (100%)	25 (100%)	25 (100%)	25 (100%)	25 (100%)	25 (100%)	≥90%	100%
Invasive cancer cases with staging and pathological dimensions N (%)	200/200 (100%)	25 (100%)	25 (100%)	25 (100%)	25 (100%)	25 (100%)	25 (100%)	25 (100%)	25 (100%)	≥90%	100%
Invasive cancer cases with state HER2 receptors N (%)	200/200 (100%)	25 (100%)	25 (100%)	25 (100%)	25 (100%)	25 (100%)	25 (100%)	25 (100%)	25 (100%)	≥90%	100%
Invasive cancer cases with state Ki67 value N (%)	200/200 (100%)	25 (100%)	25 (100%)	25 (100%)	25 (100%)	25 (100%)	25 (100%)	25 (100%)	25 (100%)	≥90%	100%
Invasive cancer cases with reported peritumoral vascular invasion N (%)	200/200 (100%)	25 (100%)	25 (100%)	25 (100%)	25 (100%)	25 (100%)	25 (100%)	25 (100%)	25 (100%)	≥90%	100%
Invasive cancer cases with reported minimum distance from the free margin N (%)	200/200 (100%)	25 (100%)	25 (100%)	25 (100%)	25 (100%)	25 (100%)	25 (100%)	25 (100%)	25 (100%)	≥90%	100%
Intraductal cancer cases with histological type N (%)	80/80 (100%)	10 (100%)	10 (100%)	10 (100%)	10 (100%)	10 (100%)	10 (100%)	10 (100%)	10 (100%)	≥90%	100%
Intraductal cancer cases with grading N (%)	73/80 (91%)	10 (100%)	**8** **(80%)**	10 (100%)	10 (100%)	**8** **(80%)**	10 (100%)	**7** **(70%)**	10 (100%)	≥90%	70–100%
Intraductal cancer cases with pathological dimensions N (%)	**67/80** **(83%)**	10 (100%)	**4** **(60%)**	10 (100%)	10 (100%)	**5** **(50%)**	10 (100%)	**8** **(80%)**	10 (100%)	≥90%	50–80%
Intraductal cancer cases with reported minimum distance from the free margin N (%)	**49/80** **(61%)**	9 (90%)	**0** **(0%)**	10 (100%)	10 (100%)	**5** **(50%)**	**0** **(0%)**	**5** **(50%)**	10 (100%)	≥90%	0–100%
Intraductal cancer cases with reported comedonic necrosis N (%)	80/80 (100%)	10 (100%)	10 (100%)	10 (100%)	10 (100%)	10 (100%)	10 (100%)	10 (100%)	10 (100%)	≥90%	100%
Invasive cancer cases with preoperative magnetic resonance imaging N (%)	92/200 (46%)	8 (32%)	10 (40%)	15 (60%)	8 (36%)	20 (80%)	3 (12%)	16 (64%)	13 (52%)	≥5%	12–80%
X-ray of the piece in 2 perpendicular projections N (%)	**134/173** **(77%)**	22 (100%)	21 (100%)	**22** **(50%)**	21 (100%)	**23** **(70%)**	**0** **(0%)**	21 (100%)	22 (100%)	≥90%	0–100%

**Table 2:** Senonetwork indicators regarding waiting time and treatment among 8 breast units. The indicators below the minimum quality threshold are in bold.

		Breast Unit									
Waiting Period and Treatment	Total	1	2	3	4	5	6	7	8	Minimum Standard	Range
Start of treatment within 30 days since therapeutic indication N (%)	**209/280** **(74%)**	33 (94%)	33 (94%)	**24** **(68%)**	35 (100%)	**18** **(52%)**	31 (88%)	**14** **(40%)**	**21** **(60%)**	≥75%	40–100%
Start of treatment within 42 days since the first diagnostic examination N (%)	**173/280** **(62%)**	33 (94%)	33 (94%)	**8** **(23%)**	35 (100%)	**14** **(40%)**	**15** **(43%)**	**14** **(40%)**	**21** **(60%)**	≥75%	23–100%
Start of treatment within 60 days since the mammography N (%)	**125/280** **(44%)**	**11** **(31%)**	28 (80%)	**8** **(23%)**	**21** **(60%)**	**14** **(40%)**	**14** **(40%)**	**10** **(28%)**	**19** **(54%)**	≥75%	23–80%
Invasive cancer single-surgery (excluding any reconstructive interventions) N (%)	157/173 (91%)	20 (91%)	21 (95%)	22 (100%)	18 (90%)	19 (100%)	20 (100%)	19 (100%)	18 (90%)	≥80%	90–100%
Non-invasive cancer single-surgery (excluding any reconstructive interventions) N (%)	65/70 (93%)	**5** **(62%)**	9 (100%)	10 (100%)	**7** **(77%)**	9 (100%)	9 (100%)	8 (100%)	8 (100%)	≥80%	62–100%
At least 10 lymph nodes in the axillary dissection N (%)	11/13 (84%)	2 (100%)	1 (100%)	2 (100%)	2 (66%)	NA	NA	3 (100%)	1 (50%)	≥80%	50–100%
Examination of sentinel lymph node(s) in only pN0 N (%)	178/187 (95%)	23 (100%)	24 (100%)	23 (100%)	21 (91%)	22 (88%)	25 (100%)	20 (91%)	20 (87%)	≥80%	87–100%
Absence of axillary dissection in non-invasive cancer N (%)	80/80 (100%)	10 (100%)	10 (100%)	10 (100%)	10 (100%)	10 (100%)	10 (100%)	10 (100%)	10 (100%)	≥90%	100%
Presence of a maximum 3 lymph nodes in SN biopsy N (%)	171/178 (95%)	21 (91%)	24 (100%)	20 (87%)	21 (100%)	22 (100%)	23 (92%)	20 (100%)	20 (100%)	≥80%	87–100%
Conservative surgery for invasive carcinoma ≤ 3 cm N (%)	161/170 (95%)	19 (100%)	20 (100%)	21 (100%)	20 (91%)	20 (91%)	23 (100%)	20 (100%)	18 (78%)	≥70%	78–100%
Conservative surgery for in situ carcinoma ≤ 2 cm N (%)	64/64 (100%)	8 (100%)	7 (100%)	7 (100%)	6 (100%)	7 (100%)	10 (100%)	10 (100%)	9 (100%)	≥80%	100%
Conservative post-surgery radiotherapy N (%)	224/234 (96%)	28 (100%)	25 (89%)	25 (92%)	28 (100%)	26 (90%)	32 (100%)	30 (100%)	30 (94%)	≥80%	89–100%
Post mastectomy radiotherapy in pN2a cases N (%)	4/4 (100%)	1 (100%)	NA	1 (100%)	NA	NA	NA	1 (100%)	1 (100%)	≥80%	100%
Radiotherapy within 12 weeks from surgery if adjuvant CT is not requested N (%)	141/160 (88%)	19 (100%)	20 (100%)	18 (100%)	15 (83%)	16 (80%)	18 (90%)	18 (90%)	17 (89%)	≥80%	80–100%
Adjuvant hormone therapy if it is an endocrine-sensitive invasive cancer N (%)	175/180 (97%)	21 (91%)	24 (100%)	22 (100%)	20 (95%)	22 (100%)	22 (100%)	22 (96%)	22 (100%)	≥80%	91–100%
Adjuvant chemotherapy if it is a hormone receptor negative invasive cancer N (%)	20/21 (95%)	2 (100%)	1 (100%)	3 (100%)	4 (100%)	3 (100%)	3 (100%)	2 (100%)	3 (75%)	≥80%	75–100%
Trastuzumab in cases treated with chemotherapy in invasive cancer N+ or HER2+ (N neg and T > 1 cm) N (%)	16/16 (100%)	3 (100%)	2 (100%)	2 (100%)	1 (100%)	NA	2 (100%)	3 (100%)	3 (100%)	≥80%	100%

NA: non-applicable.

**Table 3:** Senonetwork indicators regarding aesthetic and functional quality indicators among 8 breast units. The indicators below the minimum quality threshold are in bold.

		Breast unit									
Aesthetic and Functional Quality Indicators	Total	1	2	3	4	5	6	7	8	Minimum Standard	Range
Absence of retracting or diastasated scar N (%)	228/280 (81%)	32 (92%)	31 (88%)	31 (88%)	30 (86%)	**20 (60%)**	**23 (65%)**	32 (92%)	29 (82%)	≥80%	60–92%
Absence of skin discoloration N (%)	258/280 (92%)	33 (94%)	33 (94%)	31 (88%)	28 (80%)	33 (94%)	34 (97%)	35 (100%)	31 (88%)	≥80%	31–91%
Patients with nipple–areola complex asymmetry (conservative surgery) N (%)	127/243 (52%)	15 (50%)	13 (43%)	16 (50%)	20 (66%)	14 (43%)	18 (60%)	14 (48%)	17 (56%)	Not available	43–66%
Skin-sparing or nipple-sparing mastectomy	23/37 (59%)	**2/5** **(40%)**	4/5 (80%)	2/3 (66%)	**2/5** **(40%)**	**1/3** **(33%)**	5/5 (100%)	**2/6** **(33%)**	4/5 (80%)	≥50%	33–100%
Mastectomy with immediate reconstruction N (%)	13/23 (56%)	2 (100%)	3 (75%)	**0** **(0%)**	2 (100%)	**0** **(0%)**	**4** **(0%)**	1 (50%)	**1** **(20%)**	≥50%	0–100%
Immediate reconstruction without direct contact with the prothesis and flap N (%)	**11/13** **(84%)**	2 (100%)	**2** **(66%)**	NA	2 (100%)	NA	**3** **(75%)**	1 (100%)	1 (100%)	≥95%	66–100%
Use of acellular dermal matrix in the case of mastectomy and reconstruction N (%)	**9/23** **(39%)**	**0** **(0%)**	4 (100%)	2 (100%)	**0** **(0%)**	**0** **(0%)**	**0** **(0%)**	**1** **(50%)**	**2** **(50%)**	≥95%	69–100%
Oncoplastic surgery discussed at the multidisciplinary meeting N (%)	280/280 (100%)	35 (100%)	35 (100%)	35 (100%)	35 (100%)	35 (100%)	35 (100%)	35 (100%)	35 (100%)	≥90%	100%
Oncoplastic surgery discussed with pre and post photos N (%)	**51/280** **(18%)**	**0** **(0%)**	**0** **(0%)**	**0** **(0%)**	**8** **(22%)**	**15** **(42%)**	**10** **(28%)**	**0** **(0%)**	**18** **(51%)**	≥90%	0–42%
Percentage of lost implantation at 6 months from the immediate reconstruction N (%)	**3/23** **(13%)**	0 (0%)	**1** **(25%)**	**1** **(50%)**	0 (0%)	0 (0%)	0 (0%)	0 (0%)	**1** **(25%)**	≤9%	0–50%
Axillary dissection with homolateral axillary lymphedema N (%)	2/13 (15%)	0 (0%)	0 (0%)	1 (50%)	0 (0%)	NA	NA	1 (33%)	0 (100%)	≤20%	0–50%
Sentinel lymph node biopsy with homolateral axillary lymphedema N (%)	2/187 (1%)	0 (0%)	0 (0%)	2 (9%)	0 (0%)	0 (0%)	0 (0%)	0 (0%)	0 (0%)	≤5%	0–9%
Operated cases with articular limitations on the homolateral shoulder ≥ 10% compared to the controlateral shoulder N (%)	13/280 (5%)	2 (6%)	6 (17%)	2 (6%)	1 (3%)	0 (0%)	0 (0%)	2 (6%)	0 (0%)	≤10%	87–100%

NA: non-applicable.

## Data Availability

Data used in this study are available from the corresponding author upon reasonable request.
